# Molecular modelling and docking of Mus musculus HMGB1 inflammatory protein with CGA

**DOI:** 10.6026/97320630015467

**Published:** 2019-07-31

**Authors:** Alok Tripathi, Kriti Shrinet, Vinay Kumar Singh, Arvind Kumar

**Affiliations:** 1 School of Biotechnology, Institute of Science, Banaras Hindu University, Varanasi-221005,India

**Keywords:** HMGB1, CGA, modelling, docking, cancer

## Abstract

Recently, High Mobility Group Box1 (HMGB1) protein has been reported as an inflammatory cytokine present in all nucleated cells with crucial role in the genesis and promotion of cancer. No HMGB1 protein mice model and its active site details are available to validate mice in vivo experiments. Here, for the first time we have reported in silico mice HMGB1 model using human HMGB1 template. Prepared HMGB1 secondary structure showed 6-α helices, 5-β turns, 2-γ turns with 67% α-helices, 32% coil and 9% turn without β-sheet, and classified as α-class protein. Ramachandran plot analysis showed 98.2% and 92.3% residues lies in favoured region, verified by RAMPAGE and PDBsum server respectively. Cancer atlas of HMGB1 protein showed up-regulated expression of HMGB1 gene in different cancer, proved by CAB (CAB005873) and HPA-antibody (HPA003506) in silico. HMGB1 protein showed interaction with different biologically important inflammatory protein as depicted in STRING result.Prominent active site has residues Tyr^78^Ile^79^Pro^80-81^Lys^82^Gly^83^vGlu^84^Thr^85^Lys^86-88^Phe^89^Lys^90^Asp^91^Pro^92^Asn^93^Tyr^162^Lys^165^ with 310 Å^3^ site volume.Interacting residues of CGA-HMGB1 docked complex were ILE^79^PRO^80-81^LYS^82^GLY^83^GLU^84^LYS^86-88^PHE^89^Arg^163^Ala^164^LYS^165^Gly^166^ with docking score 3872 and surface area 412.6. CGA-conformer C3950 showed best docking than CGA and conformer-ZINC03947476, iso-chlorogenic acid and cischlorogenic acid. HMGB1 mice model could be a good therapeutic target for anti-cancerous drugs.

## Background

Cancer is a foremost cause of global death and recently, HMGB1 has been recognized for inflammation related cancer genesis [1,2]. It is made up of three domains, A-box (N-terminal domain), Bbox (central domain) and terminal C-domain [3]. Active secretion of HMGB1 occurs from immune cells e.g. macrophages, monocytes, NK cells, while passive secretion occurs from damaged necrotic cells [1,4]. It has extracellular activities as a cytokine, since mediates inflammation, proliferation and migration in different cancers [5]. Up-regulation of HMGB1 is associated with the hallmarks of cancer and clinically it has crucial role in the autoimmune diseases, apart from cancer [6-8]. Phenolics are natural antioxidant obtained from plants one of them is chlorogenic acid (CGA), naturally present in coffee, apple, mulberry, Achyranthes aspera etc [9]. CGA has anti type-2 diabetes mellitus, antioxidant activity, anti-inflammatory and anti-carcinogenic property [10,11]. Biologically CGA checks the growth and proliferation of cancerous cells the reason of which is still unknown and need to be proved experimentally [12]. Here we reported that CGA binds with the active site of HMGB1 as proved by molecular docking experiment, thus mitigating its activity and ability to cause cancer. The therapeutic molecule HMGB1 could be targeted by CGA-conformers or other biomolecules drugs to cure and prevent cancer, as our in silico data revealed.

## Methodology

### Molecular structure characterization and modelling:

The three dimensional (3D) structure of mice HMGB1 protein was not available in PDB database, hence an attempt has been made to determine the 3D structure of mice HMGB1 protein based on homology modelling. Human HMGB1 protein structure was used for characterization of mice HMGB1 protein model using BLASTp algorithm. Characterized mice HMGB1 protein sequence was used for modelling and visualization by Discovery Studio 3.0 software [13].

### Model quality assessment and verification:

Structural assessment and verification of predicted HMGB1 protein model was performed by RAMPAGE and PDBsum server [14]. Verified mice HMGB1 protein model was deposited in Protein Model Database (PMDB) [15].

### Status of HMGB1 protein expression in different cancer:

Human Protein Atlas Database (HPAD) has expression level of different cancer causing genes of interest in 20 most commonly occurring cancers [16]. Expression level of HMGB1 protein was checked in different cancers by using HPA003506 (SIGMAALDRICH) and CAB005873 (ABCAM-PLC) antibodies.

### Protein-protein interactions:

STRING (Search Tool for the Retrieval of Interacting Genes) server was used to identify the function of HMGB1 protein based on direct and indirect physical as well as functional proteinprotein interaction network [17].

### MeSH (Medical Subject Headings) classification for CGA conformer's identification:

PubChem classification browser was used for the MeSH (Medical Subject Headings) tree classification of CGA for identification of conformers [18]. Substances resulted from classification browser search results were used for the phylogeny tree preparation using structural clustering tool of PubChem database. The compounds were clustered together based on the 3D Tanimoto structure using single linkage algorithm. Representative candidate structure from each hierarchy level was selected for further preparation of closest possible phylogeny tree using structural clustering.

### Active site identification:

The Q-site Finder server based on interaction energy calculation between the protein and Vander walls probe, was used for the identification of ten prominent active sites of prepared model. Docking scores and active site volumes for each predicted active sites were also predicted [19].

### Molecular docking and docking complex visualization:

Molecular docking calculation was performed by PatchDock server and algorithm was based on shape complementarity principle. This method utilises protein-ligand molecule complexes during the docking process [20]. CGA molecule (CID- 1794427) and other conformers obtained by structure clustering approach were used for docking, complex preparation and selection for best docked complex. Docked complex of HMGB1- CGA was visualized by Discovery studio 3.0.

## Results

### Molecular structure characterization and modelling:

**Figure 1:**
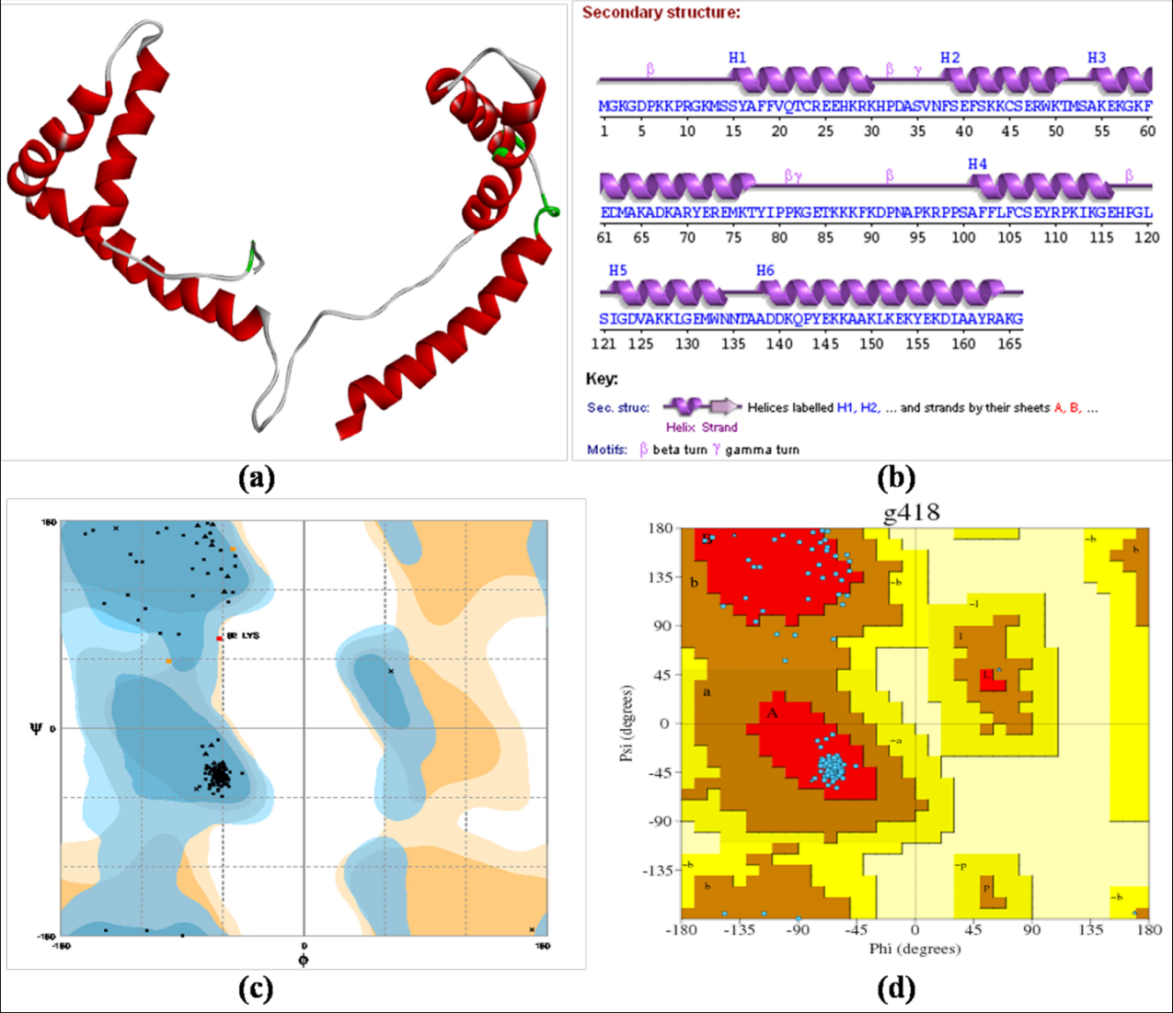
(a) Mice HMGB1 protein model generated by homology modelling approach (PMDB ID: PM0079141); (b) PDBsum wiring diagram representation of secondary structure elements containing 6-α helices, 5-β turns, and 2-γ turns; (c) Structural assessment and verification by RAMPAGE server (d) Structural assessment and verification by PDBSum server.

Predicted mice HMGB1 sequence (Accession ID-BAE29962.1) showed 99 % identity and 77% query cover with human HMGB1 protein sequence (PDBID: 2YRQ). Only A-chain sequence i.e. 2YRQ: A of template (human) was utilized for target (mice) sequence prediction [21]. Predicted protein model contains 6-α helices, 5-β turns, 2- γ turns without β-sheet and hence it was classified as α-class protein (Figure 1a). The structural composition of mice HMGB1 protein model was confirmed by the PDBsum server (Figure 1b).

### Model quality assessment and verification:

According to RAMPAGE server analysis 98.2% residues were lies in favoured region, 1.2% residues were lies in allowed region and 0.6% residues were in outlier region (Figure 1c). However, PDBsum server analysis showed that most favoured regions have 92.3% residues and additional allowed regions were having 7.7% residues (Figure 1d). PMDB-ID of mice HMGB1 protein model submitted to PMDB database is PM0079141.

### HMGB1 protein expression in different cancer by Human Protein Atlas Database:

**Figure 2:**
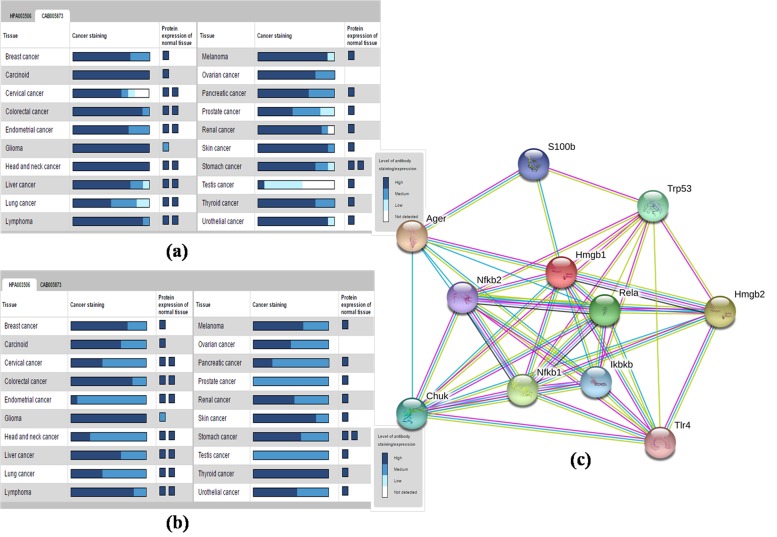
(a) Detection of expression level of HMGB1 in different cancer using CAB antibody; (b) Detection of expression level of HMGB1 in different cancer using HPA antibody; (c) Protein-protein interactions assessment by STRING database.

The cancer atlas of HMGB1 protein showed that the expression level was highest in carcinoid, glioma, head and neck cancer, while lowest in testis cancer detected with CAB-antibody (CAB005873) in silico (Figure 2a). However, expression level of HMGB1 protein was highest in glioma and thyroid cancer, while lowest in prostate and testis cancer detected with HPA-antibody (HPA003506) in silico (Figure 2b).

### STRING database for protein-protein interactions:

STRING database results showed a strong networking with reference to protein-protein interaction, depicting HMGB1 protein capability to interact with different biologically important proteins (Figure 2c). Predicted functional partners of HMGB1 protein were AGER (Advanced glycosylation end product-specific receptor), HMGB2 (High Mobility Group Box 2), NF-κB1 (nuclear factor of kappa light polypeptide gene enhancer in Bcells1), RELA (v-rel reticulo endotheliosis viral oncogene homolog A), Trp53 (transformation related protein 53), Chuk (conserved helix-loop-helix ubiquitous kinase), IκBKβ (inhibitor of kappa-B kinase-β), S100b (S100 protein, -β polypeptide, neural), NF-κB2 (nuclear factor of kappa light polypeptide gene enhancer in B-cells2) and TLR4 (Toll-like receptor4).

### MeSH (Medical Subject Headings) classification for CGA conformer's identification:

**Figure 3:**
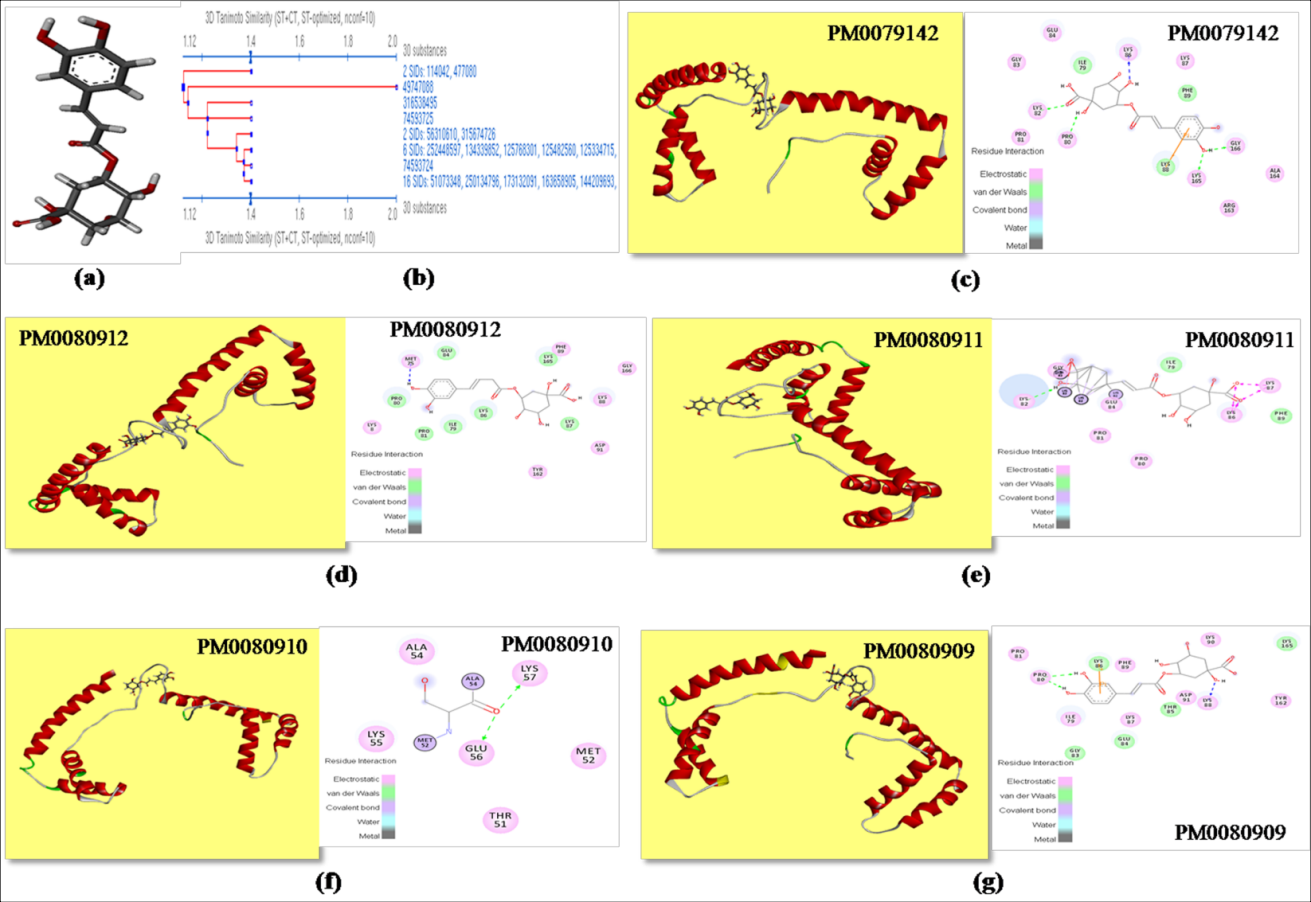
(a) Structure of chlorogenic acid used as a ligand for molecular docking (CID-1794427); (b) Identification of CGA conformers by structure clustering approach; (c) Molecular Docking of CGA with mice HMGB1 protein (PMDBID: PM0079142) and representation of active site residues and force of attractions involved in docking; (d) Molecular Docking of conformer C3950 with mice HMGB1 protein (PMDB ID: PM0080912) and representation of active site residues and force of attractions involved in docking; (e) Molecular Docking of conformer ZINC03947476 with mice HMGB1 protein (PMDB ID: PM0080911) and representation of active site residues and force of attractions involved in docking; (f) Molecular Docking of conformer iso-chlorogenic acid with mice HMGB1 protein (PMDB ID: PM0080910) and representation of active site residues and force of attractions involved in docking; (g) Molecular Docking of conformer cis-chlorogenic acid with mice HMGB-1 protein (PMDB ID: PM0080909) and representation of active site residues and force of attractions involved in docking.

Total one hundred substances were obtained for CGA (CID- 1794427; Figure 3a), a ligand molecule from PubChem classification browser search. Structural similarity algorithm was applied for structure clustering of resultant substances and ninety eight structures were clustered in eight level of hierarchy (Figure 3b). One substance from each hierarchy level was selected as representative substance because same hierarchy level substances considered as a same substance. Most importantly substance with SID-211535102, 196107032 and 316538495 were showing common CID-1794427 which is similar to the core ligand molecule CGA. Remaining four substances with CID-1794425 (cis-chlorogenic acid), 24802030 (C3950), 11870309 (ZINC03947476) and 5315832 (iso-chlorogenic acid) were used as final conformers of CGA which was selected for the further in silico studies.

### Active site identification:

Total ten active sites were predicted by Q-site Finder server for prepared HMGB1 protein model to decipher the docking of CGA ligand. The first active site was most prominent and suitable for any ligand binding due to highest site volume. The prominent active site has Tyr^78^Ile^79^Pro^80^Pro^81^Lys^82^Gly^83^Glu^84^Thr^85^ Lys^86^Lys^87^Lys^88^Phe^89^ Lys^90^Asp^91^Pro^92^Asn^93^ Tyr^162^Lys^165^ AAs residues.

### Molecular docking of ligand CGA with HMGB1 protein:

CGA molecule (CID-1794427) showed 3872 docking score with surface area 412.6 (Table 1). Visualization of docked complex (HMGB1-CGA) showed interaction of Ile^79^Pro^80^Pro^81^Lys^82^Gly^83^ Glu^84^Lys^86^Lys^87^Lys^88^Phe^89^Arg^163^Ala^164^Lys^165^Gly^166^ residues of HMGB1 prominent active site with ligand CGA (Table 1). Docked HMGB1-CGA model was successfully submitted to PMDB database with generated PMDB-ID PM0079142 (Figure 3c). Docking of selected conformers of ligand CGA molecule was also done with prominent active site of HMGB1 protein, to find out the extent of their comparative stability. The conformers docked model were successfully deposited to PMDB database with generated PMDB-ID for C3950 conformer (CID-24802030) PM0080912 (Figure 3d), for ZINC03947476 conformer (CID-11870309) PM0080911 (Figure 3e), for iso-chlorogenic acid conformer (CID-5315832) PM0080910 (Figure 3f), and for cis-chlorogenic acid conformer (CID-1794425) PM0080909 (Figure 3g). Docking complex prepared with conformer C3950 (CID-24802030) showed best docking score and surface area interaction value 4296 and 508.4 respectively and residues involved in the interaction are Met^75^Ile^79^Pro^80-81^Lys^82^Glu^84^Lys^86- 88^Phe^89^Asp^91^Tyr^162^Lys^165^Gly^166^ (Table 1).

## Discussion

Sequences used for homology modelling of mice HMGB1 protein showed 99% structural identity and 77% query cover with human HMGB1. This was to confirm that first ever generated mice HMGB1 model is as good as that of reference human HMGB1 model for further experiments. According to Ramachandran, predicted protein structures could be acceptable if it contained overall high percentage of Φ and Ψ values within allowed range. Our results of Ramachandran plot generated by RAMPAGE and PDBsum servers showed that percent residues were maximum lied in favoured region and none of the residues were in the disallowed region, indicated that the protein model is of good quality. Expression of HMGB1 protein was observed upregulated in most of the cancers as shown in generated cancer atlas result (Figure 2a,Figure 2b),confirming that HMGB1 protein could be targeted in various types of cancers. HMGB1 structure plays various key roles by auto up-regulated expression as cytokines to activate immune cells, many inflammatory cytokine genes and also as TFs to bind and up-regulates the responsible cancer genes, observed by in silico study using CAB and HPA antibody (Figure 2a, Figure 2b). HMGB1 plays an important role by protein-protein interaction with TLR-4, NF-κB, and other TFs e.g. STAT and thus involved in the activation of inflammatory pathway [22]. The activity of HMGB1 protein got inhibited was proved by docking experiments, where inhibitor molecule CGA binds to its active site (Figure 3c). Surprisingly, all four docking complex showed better docking score as well as docking area in comparison to initially docked CGA molecule (Table 1). Docking result with all CGA molecules conformer was good but C3950 conformer docked complex was found best and stable. By using this approach anyone could predict the conformer of any ligand molecule which showed best docking with selected target.

## Conclusion

Overall, our finding based on results of HMGB1 protein structure model is very trustworthy, first ever report in mice and could be utilized for the docking as well as prediction of CGA, CGAconformers like natural biomolecule drugs that should bound to the prominent active target site of cancer causing inflammatory cytokine HMGB1 to prevent and cure several types of cancer. The designed mice HMGB1 protein model and HMGB1-CGA in silico docking model might be path breaking finding in the discovery of potential universal anticancer drug effective against various cancer types.

## Figures and Tables

**Table 1:** Submitted PMDB-ID of docked complex with docking score, surface area and interacting residues of active site.

S. No.	Ligand used for docking	PMDB-ID of docked complex	Docking Score	Surface area	Residues involved in docking	Common residues in All docking model
1	CID 1794427 (CGA)	PM0079142	3872	412.6	Ile79, Pro80, Pro81, Lys82, Gly83, Glu84, Lys86,Lys87, Lys88, Phe89, Arg163, Ala164, Lys165 and Gly166 (14 residues)	
2	CID 24802030 (C3950)	PM0080912	4296	508.4	Met75, Ile79, Pro80, Pro81, Lys82, Glu84, Lys86,Lys87, Lys88, Phe89, Asp91, Tyr162, Lys165 and Gly166 (14 residues)	Ile79, Pro80, Pro81, Glu84, Lys86, Lys87, Phe89 (7 residues)
3	CID 11870309	PM0080911	4222	474.3	Ile79, Pro80, Pro81, Lys82, Gly83, Glu84, Lys86, Lys87 and Phe89 (9 residues)	
4	CID 5315832	PM0080910	4162	495.2	Thr51, Met52, Ala54, Lys55, Glu57 and Lys57 (6 residues)	
5	CID 1794425	PM0080909	3916	424.1	Ile79; Pro80, Pro81, Gly83,Glu84, Thr85, Lys86,Lys87, Lys88, Phe89,Lys90, Asp91, Tyr162 and Lys165 (14 residues)
